# The framework and best practices of emergency medical teams: lessons learned from Israel’s international field hospital response

**DOI:** 10.1186/s13584-026-00751-6

**Published:** 2026-03-25

**Authors:** Evan Avraham Alpert, Giora Weiser, Deganit Kobliner-Friedman, Mitchell J. Schwaber, Ami Mayo, Reuven Kedar, Nehemia Blumberg, Ofer Merin

**Affiliations:** 1 Department of Emergency Medicine, Hadassah University Medical Center-Ein Kerem, Jerusalem, Israel; 2 Faculty of Medicine, Hebrew University of Jerusalem, Jerusalem, Israel; 3 Department of Pediatric Emergency Medicine, Shaare Zedek Medical Center, Jerusalem, Israel; 4 Department of Emergency Medicine, Shaare Zedek Medical Center, Jerusalem, Israel; 5 National Center for Infection Control, Israel Ministry of Health, Jerusalem, Israel; 6 Department of Medicine, Gray Faculty of Medicine and Health Sciences, Tel Aviv University, Tel Aviv, Israel; 7 Intensive Care Department, Samson Assuta Ashdod Medical Center, Ashdod, Israel; 8 Faculty of Medicine, Ben-Gurion University of the Negev, Beer Sheva, Israel; 9 Department of Obstetrics and Gynecology, Genetics Institute, Carmel Medical Center, Haifa, Israel; 10 The Ruth and Bruce Rappaport Faculty of Medicine, Technion - Israel Institute of Technology, Haifa, Israel; 11 Shaare Zedek Medical Center, Jerusalem, Israel

**Keywords:** Disasters, Earthquakes, Field hospital, Israel, World Health Organization

## Abstract

In 2010, a devastating earthquake struck Haiti, killing over 200,000 and injuring over 250,000. Dozens of countries sent medical teams, with Israel’s being one of the first to arrive. These teams saved lives and salvaged limbs. However, other responses were not as professional, resulting in criticism in the medical community of “Disaster Tourism.” For example, certain physicians attempted to perform procedures or surgeries that they were not certified to perform. There was a call to develop a framework for disaster response. The World Health Organization spearheaded this project by initiating the formation of Foreign Medical Teams, which later became known as Emergency Medical Teams (EMTs). The EMT Initiative involves a vision of saving lives in the context of transparency and global cooperation. It includes an emphasis on safe care, ethical care, and an accountable, coordinated response to disasters. There are three general types of teams, with an additional group of specialized care teams. An EMT-1 is similar to a clinic and open only during the day. An EMT-2 adds operating room and inpatient capabilities. EMT-3 which is the most complex, includes a minimum of 40 inpatient beds, four intensive care unit (ICU) beds, and the capacity to treat 100 outpatients a day. The Israel Defense Forces Field Hospital was the first in the world to be recognized as an EMT-3. The Israeli EMT-3 includes an emergency department, inpatient services, surgical capabilities, and an intensive care unit. Pediatric services, infectious disease care, and women’s health are integral parts of the EMT-3. Logistical and ancillary services also play a key role in the success of the field hospital. The Israeli field hospital responded to recent disasters in Haiti (2010), the Philippines (2013), Nepal (2015), and Turkey (2023). The main lesson learned is that the success of the Israeli EMT-3 is based on professionalism and flexibility within the framework of the EMT initiative. The staff consists of highly trained professionals, including board-certified physicians and nurse specialists who convert their everyday skills into those used in the disaster setting. Flexibility includes having these professionals take on logistical responsibilites. Operational flexibility includes the last-minute decision to implement a hybrid versus a standalone model, to using local supplies to quickly adapt to an urgent need. While the lessons here are from the perspective of an EMT-3, they can be applied to other response teams to improve outcomes for disaster victims throughout the world.

## Background

The earthquake that devastated Port-au-Prince, Haiti, in 2010 served as a turning point in international medical disaster response. The magnitude of the destruction and the degree of need for urgent assistance led to the rapid arrival of medical personnel from throughout the world. In some cases, such as that of Israel, these were teams sent by a country [1]. In others, they were sent by a medical institution or volunteer organization, and in still others, they consisted of individuals or small groups acting on their own behalf. While lifesaving work was performed in many cases by these teams, the absence of a central body charged with assigning certifiably qualified and accountable teams to areas of need in a coordinated manner was acutely felt [2, 3].

Arising from the experience of Haiti, there was a call to develop a framework for disaster response. International meetings took place in December 2010 in Cuba, organized by the Karolinska Institute and the Pan American Health Organization/World Health Organization (WHO), and in April 2011 in Stockholm, to develop guidelines for foreign field hospitals (FH) and standards for surgical care in disaster response [4].

As a result, the WHO initiated the project known as Foreign Medical Teams, which later became known as Emergency Medical Teams (EMT). The EMT Initiative involves a vision of saving lives in the context of transparency and global cooperation. It includes an emphasis on safe care, ethical care, and an accountable, coordinated response to disasters. It involves an eight-step pathway that includes a self-assessment, mentorship, verification visit, and international registration. There are multiple core standards that include coordination with other teams, training, record keeping, professional conduct, self-sufficiency, and operational management. There are three general types of teams, with an additional group of specialized care teams. An EMT-1 is similar to a medical clinic and is divided into a mobile and fixed type. The mobile EMT-1 has the capacity to see at least 50 outpatients a day during daytime hours, with the Fixed EMT-1 able to see 100 outpatients a day with an on-call back up team. The EMT-2 adds operating room capabilities and must be open 24/7. This includes the ability to perform seven major or 15 minor operations per day, a minimum of 20 inpatient beds, and the ability to treat 100 patients per day. The EMT-3 is known as a complex referral-level inpatient center with the ability to perform 15 major and 30 minor operations per day. It must have a minimum of 40 inpatient beds, four intensive care unit (ICU) beds, and an emergency department (ED) with the facilities to treat 100 patients a day. In addition, the fourth type of EMT, specialized care teams, focus on specific needs such as for advanced burn care or rehabilitation [5].

There are the “Four S” of EMTs that emphasize system, staff, supplies, and structure/space. In addition, there are minimum standards which are part of the formal standardization mechanism established under the EMT initiative. This includes triage, assessment, resuscitation, stabilization, referral, and transfer. There are also patient-specific standards related to wound care, burn care, fracture care, managing communicable diseases, reproductive health, pediatrics, analgesia, anesthesia, intensive care, surgery, malnutrition, palliative care, rehabilitation, mental health, transfusion services, laboratory services, imaging, pharmacy, and sterilization. There are nonmedical standards related to communication, transportation, food, warehouse management, facility structure, sanitation, pest control, dead body management, and donations [5].

In 2025 there were 55 EMTs worldwide [6]. Most of these are EMT-1 or EMT-2. In 2016, the Israel Defense Forces (IDF) became the first certified EMT-3. Later in 2018, Sichuan, China also became an EMT-3 [7]. There have been many publications about individual field hospital (FH) missions [8–10] as well as articles based on specific Israeli EMT-3 deployments to past disasters [1, 11–13]. The objective of this article is to describe the framework and best practices of an EMT-3 as learned from a comprehensive review of Israel’s international FH response. These lessons are based on debriefings that occurred during and immediately after the deployments. During each mission there were evening meetings with all department directors, and a debriefing with all delegation members at least once during, and then at the end of the deployment. The department heads then presented a post-deployment written summary to the medical directors.

## Main Text

### History of the Israel defense forces field hospitals

#### Early history

The first IDF humanitarian medical mission was deployed in 1953 in response to an earthquake in Greece. This was a mobile clinic staffed by physicians and medics. Over the next two decades, several other delegations were sent to earthquakes in Macedonia (1963), Mexico (1985), and Cameroon (1986). The first time that Israel sent a full FH to a disaster zone was in 1988 in response to a massive earthquake in Armenia. Since then, Israel dispatched FHs in response to wars in Rwanda in 1994 and Kosovo in 1999. Field hospitals were deployed in response to sudden-onset disasters such as earthquakes in Turkey (1999) and India (2001) [14].

#### Haiti-2010

On January 16, 89 h after the devastating earthquake struck Haiti, causing casualties in the hundreds of thousands, the IDF FH deployed on a soccer field in Port-au-Prince, in the context of a humanitarian mission that also included a search and rescue team. Given the magnitude of devastation and the incapacitation of local services, the Israeli mission was by necessity fully self-sufficient, medically and logistically. There were 121 staff members, including 44 physicians. Operating under a central hospital command, staff were divided into medical, surgical, orthopedic, pediatric, obstetrics/gynecology (OB/GYN), and ambulatory care teams. There were 60 inpatient beds, with a surge capacity of 72. Ancillary services included chemistry, hematology, and microbiology laboratories, imaging, informatics, engineering, and logistics. Specialists in infectious diseases and mental health provided consultative support. The deployment of a surgical team from the Colombian military on the grounds of the Israeli FH allowed for increased output.

During the 10 days of operation, 1,111 patients passed through the triage point, of whom 737 were hospitalized. There were 244 surgical procedures that were performed on 203 patients. Overall, 2/3 of the admissions were due to trauma, with the trauma:non-trauma ratio decreasing over time. The arrival of foreign teams and the restoration of some local services allowed the FH to conclude its mission, transferring those patients not yet ready for discharge to other providers on the scene [1, 15].

#### Phillipines-2013

On November 7, 2013, Typhoon Haiyan (Yolanda) made landfall in the Philippines at peak intensity with ten-minute sustained winds of 230 km/h. Over 5000 people were killed. The IDF-FH left for the Philippines on November 13, landing on the Island of Cebu, bringing 147 medical personnel. They joined with the 80-bed Severo Verallo Memorial District Hospital, which was usually staffed with five physicians and included an ED, a single operating room, a delivery room, a pediatric ward, maternity ward, and separate male and female medical/surgical wards. The Israeli team worked according to a fully integrative/collaborative model whereby the FH was adjacent to the local hospital. The Israeli FH was responsible for the outpatients and ancillary services, whereas the inpatients were the responsibility of the local team with the assistance of the Israeli contingent. Surgeries were performed by both teams. During their 10-day stay, they treated over 2686 patients, performed 60 surgeries, and delivered 36 babies. The team treated 848 children during the mission [16].

#### *Nepal* -2015

On Saturday, April 25, at 1156 local time, an earthquake of 7.8 on the Richter Scale occurred, killing more than 8800 and injuring more than 23,000. Israel sent a team that arrived 80 h after the earthquake. The FH was set up as a standalone hospital in tents located on the grounds of a military base. This team, which comprised an EMT-3, consisted of 124 personnel, of whom 42 were physicians. Four wards were established: pediatrics, adult medical/surgical, OB/GYN, and an ICU. The logistics sections included: command and control, laboratory, and informatics. They treated 1668 patients. These included a young male rescued 120 h after being extracted from the rubble, who was stable and discharged after 24 h of observation, and a young woman who was brought in after being rescued after 132 h under the rubble. She required multiple surgeries and a prolonged course of rehabilitation [12].

#### Turkey- 2023

On February 6, 2023, a 7.8 Mw earthquake struck eastern Turkey and Western Syria. Over 50,000 people were killed, and more than 100,000 injured. Israel deployed a medical mission with 142 members, including 58 physicians, 32 nurses, 9 logistical staff, 5 paramedics, as well as laboratory workers, radiology technicians, public health officers, and support staff. The team decided that the focus of this mission was to join the existing Necip Fazil Hospital in Kahramanmaras.

From February 9, through February 14, 2023, the Israeli EMT-3 treated 470 patients. This included eight children who were rescued from under the rubble after 100 h. One four-year-old child was rescued from the rubble after 141 h and arrived with decreased mental status and a temperature of 29 C°. All of the children were discharged in good condition. The team performed 10 operations, treated 470 patients in the ED, 27 in the ICU, and 48 in the inpatient medical/surgical ward. They cared for 17 patients who were rescued from the rubble, performed 1365 laboratory tests, 158 X-rays, and 48 dental treatments [17–19].

#### MODEX

Due to the success of the Israeli EMT-3, the team joined the European Union for a multinational disaster exercise in Romania on October 14–18, 2018. The scenario was that Romania was struck by a massive earthquake. There were 1500 participants from Romania and another 517 from 19 countries. The Israeli team included the ED, inpatient ward, laboratory, radiology, and ICU. These different departments trained and debriefed with their European colleagues. This included a simulated drill whereby there was an aeromedical transfer of patients in conjunction with the Israeli Air Force. The focus of this large-scale simulation was to learn lessons related to the coordination of international teams to improve real-world operational responses to disasters [20].

## Lessons learned

### Hospital design

A common feature of disaster relief is uncertainty. The teams may not know the extent of the damage, the number of victims, or the best site to locate the FH. The Israeli EMT-3 usually sends a small advance team prior to overall deployment to identify the best location to set up the FH. This must be in proximity to the disaster zone but also close to local medical facilities. There may be a need to either receive local patients for more advanced care or there may be resources such as advanced imaging or surgery facilities that can be used by the Israeli EMT-3. The most important factor for site selection is safety. In the setting of a natural disaster such as an earthquake, where there may be aftershocks, it is preferable for the FH not to be in a tall building and to have civil engineers verify the stability of the hospital structure. As there may be an outbreak of violence, there is a need to find a space with clear borders, that can be guarded, while at the same time, there must be an easy evacuation route. Other issues that are important for site selection include proximity to functioning electricity, water systems, or a helipad.

**Fig. 1:**
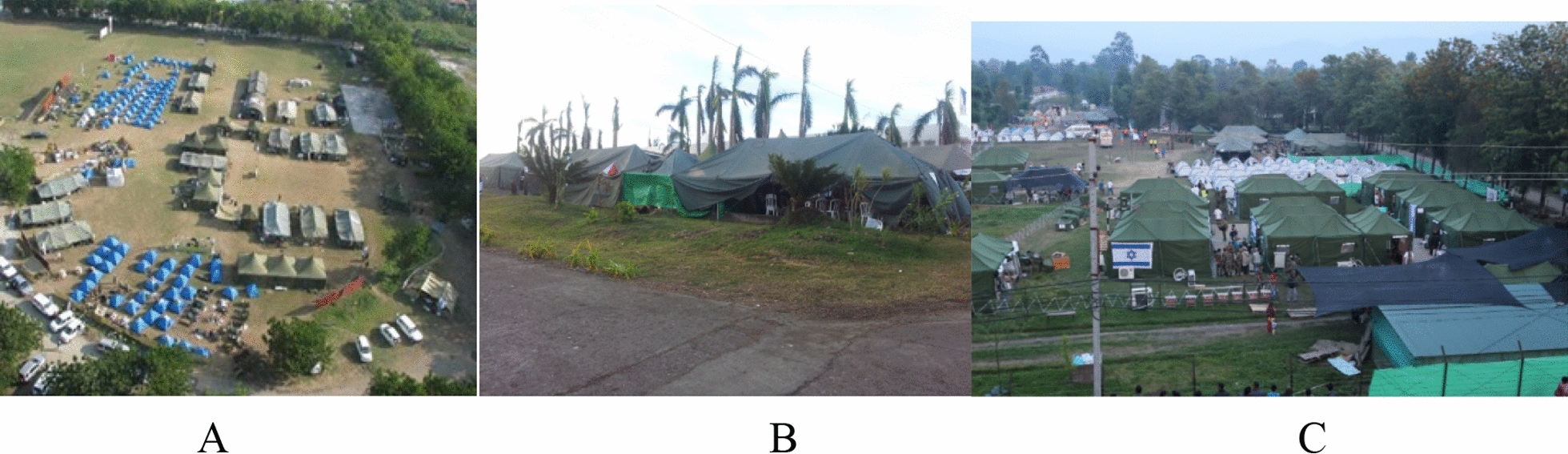
Variations in IDF field hospital design: 1**A**: Haiti; 2**B**: Philippines; 3**C**: Nepal

The Israeli EMT-3 maintains versatility in terms of setting up the hospital. Two designs are usually used- either a stand-alone or a hybrid design. The stand-alone design, as used in Haiti and Nepal, is where the tents were set up independent of existing structures. These included the ED, inpatient wards, ICU, operating rooms, and ancillary services. During the missions to the Philippines (2013) and Turkey (2023) the Israeli team joined with existing hospitals and worked with the local teams. This challenge required versatility from both a logistical and an organizational standpoint. The Israel teams needed to quickly learn how to work with local teams. Language barriers were quickly overcome with either the common language being English or the use of translators. The teams also needed to quickly adapt to the use of different equipment or local cultural nuances (see Fig. 1) [21].

### Emergency department

The ED is designed so that it can operate independently to start seeing patients within 4–6 h of arrival. Whether the hybrid or stand-alone model is used, the ED always has an ambulatory area, an acute care area, and a resuscitation zone. Pediatric patients can either be seen in a separate area or integrated with the adult patients. All standard ED supplies are brought by the team. This includes monitors, ventilators, video laryngoscopes, and portable ultrasound machines. One of the initial challenges was performing X-rays within the department, as the size of the ED is small and patients and staff would be exposed to radiation. An effort was made to purchase portable point-of-care ultrasound (POCUS) machines that are compatible with the rugged conditions of a FH. Their use includes resuscitation, procedural performance, and diagnosis [22]. There is a dedicated staff of general and pediatric emergency medicine specialists who work along with certified emergency nurses. An additional challenge is staffing, which must be 24/7. However, unlike established EDs in Israel, the trends of patient visits are unknown until the team arrives at the disaster site. One factor seems to be consistent—most patients in the disaster zone arrive during daylight hours or early evening and therefore the bulk of the staffing is during those times. However, the exact shifts in terms of hours, (whether 8 or 12) or numbers of staff per shift, are made shortly after arrival.

### Surgery and orthopedics

In natural disasters such as earthquakes, most of the patients who suffer severe abdominal, or chest injuries won’t survive. Thus, most trauma patients who will need treatment will be those with orthopedic injuries- mostly limb and spine. Those with stable pelvic fractures won’t need treatment, and in the field setup, spinal injuries can’t be treated surgically. Most of the orthopedic procedures are for soft tissue debridement, splinting, and casting. Femoral fractures are treated by external fixation, as are Gustillo 2–3 tibial fractures.

During the response to the earthquake in Haiti in 2010, of the 244 surgical procedures that were performed under general or regional anesthesia, 221 were for orthopedic injuries, mostly soft tissue debridement and wound closure. This included external fixation for 73 fractures [23]. Children were more likely than adults to undergo surgery for traumatic injuries (44% vs. 29%) [24]. The team had to be innovative, such as when they procured the help of a blacksmith to help make the proper pins for the fixation of open fractures.

In the Nepal delegation, the FH worked side by side with the local regional hospital, with an intact operating room in a much more sterile environment. Thus, all of the sterile operation, mainly closed fractures, were treated in the Nepali hospital, while all the contaminated operations were performed in one of the two FH operating theaters.

### Pediatrics

Children are disproportionately represented in public health emergencies, whether by trauma, infectious disease, or mental health issues [25]. These children will present with acute injuries, chronic illnesses, and the psychological and social effects of the disaster [26]. The appropriate pediatric team to offer solutions for this very vulnerable population should include medical staff comprised of pediatric emergency medicine, general pediatrics, and pediatric intensive care specialists. In addition, the nursing team must have either prior experience or dedicated teaching in the evaluation and treatment of children. Such a team will be able to offer the needed evaluation as well as direction for the management of pediatric cases of differing severity with their special needs and considerations [27]. In addition to a child-oriented team, the equipment necessary must be appropriate for the pediatric age group. This is a complicated issue as the ages and needs create an equipment challenge. The need for age-appropriate and pediatric-specific equipment should be decided upon long before deployment. This may include newborn incubators, endotracheal tubes for all ages, blood pressure cuffs, medications in syrup form, pediatric-specific ventilators, and intravenous pumps. These decisions have economic effects on the mission and its expectations. Improvising has also proven to be important. Examples include preparing customized fluids for premature infants from standard fluids and even creating a makeshift incubator for a newborn to enable transfer [28].

Ideally, the pediatric patients should be treated at a separate site from the adult population. This stems from a need for enabling family presence (e.g. nursing mothers) and creating a safe and “child friendly” environment. However, if unable to offer such a separation, cordoning off a pediatric section may suffice. This was implemented in the EMT deployment in Turkey and was satisfactory to both families and the medical team [17]. Working with the pediatric population also requires offering medical solutions not usually intended for disaster situations, such as the use of procedural sedation and medical clowns [29, 30].

It is important to prepare the entire EMT regarding the nuances of treating pediatric patients. This should include both general information as well as simulations and discussions of expected difficulties, such as loss of family and support, pain management, and dealing with pediatric fatalities.

### Inpatients

Inpatient wards in the FH vary with the nature of the mission. The general inpatient ward is equipped to provide care for all patients not requiring hospitalization in the ICU. Staff physicians are specialists in internal medicine, family practice, and pediatrics. Nursing tasks are performed by registered nurses. Emergency medical technicians may be incorporated in the staffing, but must limit their skills based on their level of certification, and work under the supervision of nurses. Ward rounds are conducted at specified intervals daily and ad hoc, with specialty consultation available as needed. Versatility and surge capacity are hallmarks of inpatient care in the FH setting, and vary according to the mission. In Nepal, when the initial inpatient tent was filled to capacity, an additional tent was quickly erected as another ward.

### Intensive care unit

In a FH deployed to a disaster area, the ICU plays a critical role in stabilizing and treating the most severely injured patients. The ICU provides advanced monitoring, life-support equipment, and specialized medical staff capable of managing complex trauma cases such as crush injuries, severe head trauma, respiratory failure, and multi-organ dysfunction. Its presence ensures that patients who require continuous critical care can be managed on-site rather than being immediately transferred to distant tertiary hospitals, which may be overwhelmed or inaccessible due to damaged infrastructure. By serving as a hub for advanced resuscitation and post-operative care, the ICU enhances survival rates, reduces complications, and supports the overall effectiveness of the emergency medical response in disaster conditions.

At the same time, the ICU raises profound ethical dilemmas: the very resources that can sustain one critically ill patient for days—ventilators, intensive nursing, and medications—might otherwise be used to treat dozens of moderately injured survivors. Medical teams must grapple with the tension between maximizing individual survival and optimizing collective outcomes, often making rapid triage decisions under immense pressure.

Furthermore, the ICU in a FH setting is constrained by the limited availability of advanced treatment modalities that would normally be accessible in tertiary centers, such as renal replacement therapy for acute kidney injury or extracorporeal membrane oxygenation for refractory respiratory failure. Patients who would otherwise be candidates for these life-saving therapies may receive only supportive care, forcing clinicians to confront the painful reality of providing less-than-ideal treatment due to logistical constraints. This gap not only underscores the limits of field-based critical care but also intensifies the ethical weight of triage, as providers must weigh the potential futility of care in the absence of advanced interventions against the urgent needs of many other patients who could benefit from available resources.

Given these limitations, an EMT-3 ICU must be managed by highly experienced senior intensive care specialists, both physicians and nurses, who possess the expertise to adapt clinical decision-making, optimize resource allocation, and deliver safe, effective care under austere conditions. Their leadership and judgment are critical to overcoming the constraints of limited equipment and therapies, ensuring that the ICU operates not only as a site of advanced treatment but also as a center of resilience and ethical stewardship in disaster response [31, 32]. During various deployment missions, these key principles guided the planning and integration of an ICU into the Israeli EMT-3:

- Use clear go/no-go criteria for whether or not to deploy an ICU.
- Adopt a limited, high-yield intervention set to treat more patients; combine with early, senior-led triage and end-of-life frameworks.
- Staff with highly experienced ICU physicians and nurses (24/7 attending coverage), leveraging diverse backgrounds (anesthesiology, surgery, pulmonology).
- Build modular capacity (“model of four” beds) and maintain strict nurse team structures (2 + 2 per shift) to ensure safety and continuity. This organizational concept is based on a modular “model of four,” consisting of four ICU beds functioning as a single operational unit. Each four-bed module is staffed per shift by two registered critical care nurses supported by two registered nurses. If registered nurses are unavailable, paramedics may fulfill this role under strict nursing and physician supervision. These teams function as stable organic units, with only limited cross-switching permitted. This modular structure enables straightforward scaling by doubling or tripling the number of four-bed units, provided that staff and equipment expansion is preplanned.
- Ensure environmental control (bedside air conditioning per bay) to prevent hypo/hyperthermia; monitor core temperature continuously.
- Prefer oxygen concentrators compatible with low-pressure ventilators; secure power with automatic backup systems.
- Rely on POCUS and portable x-ray; compensate for absent CT/MRI by linking to local assets.
- Standardize point-of-care labs, unique per-bed supply packs, and prepacked procedure kits to minimize errors and infections.
- Plan safe patient transport with trained teams and checklists to access external resources like imaging and dialysis.
- Embed infection control and antimicrobial stewardship tailored to local multidrug-resistant patterns.
- Maintain electronic medical records (EMR) with paper fallback.

### Obstetrics and gynecology

The OB/GYN department plays a crucial role in humanitarian missions, as demonstrated during relief operations in Haiti, the Philippines, and Nepal. These missions highlighted the unique medical needs that arise in disaster-stricken areas, where women’s healthcare often becomes critically compromised due to infrastructure collapse and limited medical resources.

During these deployments, the OB/GYN unit addresses a wide spectrum of urgent medical conditions. Emergency obstetric care represents a primary focus, including managing complicated deliveries, cesarean sections, and pregnancy-related complications that cannot be postponed despite challenging field conditions. The department also provides essential gynecological surgeries for conditions such as ovarian cysts, uterine emergencies, and trauma-related injuries that disproportionately affect women in disaster zones.

Beyond emergency interventions, the gynecological team offers critical preventive care and family planning services. This includes contraceptive counseling, prenatal care for pregnant women, and treatment of gynecological infections that often proliferate in post-disaster environments due to poor sanitation and overcrowding.

The psychological component of gynecological care proves equally vital, as female patients frequently suffer from trauma-related stress that manifests in reproductive health issues. The department’s culturally sensitive approach helps bridge communication gaps while respecting local customs and religious practices surrounding women’s healthcare.

These humanitarian missions demonstrate how specialized gynecological care in a FH extends beyond immediate medical treatment, addressing long-term reproductive health needs and contributing to community resilience. The integration of comprehensive gynecological services in emergency medical responses by the Israeli EMT-3 reflects an understanding that women’s healthcare remains essential even in the most challenging circumstances, ultimately supporting broader humanitarian objectives and population health recovery [33].

### Infection control

Infection prevention and control (IPC) in the context of a FH deployed in a disaster setting presents myriad challenges. Among the principal categories relevant to IPC are design and location, ensuring physical safety as well as adequate patient distancing, ventilation, and isolation when necessary; potable drinking water; food safety; facilitation of hand hygiene; instrument cleaning and disinfection/sterilization; location of toilets and latrines; pest control; human and solid waste disposal, including sharps; and healthcare worker safety including pre-mission vaccination and post-exposure evaluation and prophylaxis when necessary.

In the context of WHO accreditation, the Israeli EMT-3 conducted a review of its IPC protocols and underwent staff training provided by a physician specialist in infectious diseases and an infection control nurse. While the fundamental principles of IPC apply to all deployments, the unique circumstances of each individual mission dictate the specific measures to be implemented on site [34].

### Nursing

The minimum standards as discussed in the Bluebook is a nurse-patient ratio of 1:8 per shift in the ED, with higher ratios in the ICU, which is maintained in the Israeli EMT-3. The minimum nurse-physician ratio is 3:1 ; however, the Israeli EMT-3 maintains a ratio closer to 2:1 with a team approach [5, 21].

Nursing staff are military reserve-duty expert professionals drawn from leading Israeli hospitals, each a specialist in their respective departments, yet with a high-level of experience in other FH departments. The command decision to implement diffused professional leadership reflects confidence in the handpicked nursing professionals. This diffusion facilitates versatility and cross-departmental leadership. Under emergency relief conditions, nursing staff can quickly and efficiently move between departments, smoothly filling in when staffing shortages arise in the field.

We also found that diffused professional nursing leadership, under the overall chain of command discipline structure, promotes communication, self-esteem, resilience, and innovation. Nursing leaders act as front‐line guides making accurate and timely judgments regarding planning and resource allocation. Good nurse leadership empowers nurses and enables them to offer a more agile response to disasters.

### Informatics

Israel’s EMT-3 has been using an EMR since it was first utilized in Haiti in 2010. This requires at least one dedicated informatics specialist on each mission. Patients are registered into the system by name and given a specific identity number placed on a barcoded bracelet that they will use throughout their hospital stay. This barcoded bracelet can be scanned from department to department. Frontal pictures of all patients are taken and entered into the EMR. A command-and-control dashboard allows the ability to follow patient admissions, flow through the hospital, and discharge. A picture archiving and communication system (PACS) is integrated into the EMR [35]. The standard is to write the discharge summary and instructions in English. One challenge is that patients often don’t understand English. In the future, perhaps, with the rapid development of internet translation programs and artificial intelligence, the instructions can be translated into the patient’s native language.

### Ancillary services

A large contribution to the success of Israel’s EMT-3 are the ancillary services. This includes laboratory services, radiology, and sterilization. Laboratory services, staffed by professional technicians, includes basic blood tests such as a complete blood count and chemistry, but also microbiology capabilities. After the response to the earthquake in Nepal in 2015 the laboratory examined cultures from urine, wounds, sputum, and stool. Gram stains were done, and cultures were performed on selective and non-selective media. The most common bacteria that were isolated were enterobacteriaceae, but Methicillin Resistant Staphylococcus Aureus and Pseudomonas were also identified. However, blood cultures were not available [36]. A blood bank, including units of Type O blood, is brought with the team.

Radiology services are provided by at least one radiologist and one technician. The team uses a digital radiology system and PACS. Images are performed in a single radiology suite. The images can be viewed on any of the computer workstations and integrated into the patient’s EMR [37]. Portable X-rays are intentionally limited so as not to expose staff and patients to radiation. POCUS has been used extensively by emergency physicians, surgeons, and critical care clinicians and is integrated throughout the FH. Both portable and handheld machines are used.

### Logistics

Once the Israeli EMT-3 is notified of the need to respond to a disaster, the target for mobilization is 12 h. This includes the time to activate the staff and bring the supplies from storage to the airport. The team is required to be self-sufficient, bringing supplies for up to two weeks. This includes all medical supplies and food. Often, an additional delivery is made during the mission. A small staff of logistics personnel accompanies all missions. This includes at least one electrician, civil engineer, specialist in water purification, and public health expert. There are also operations staff that are involved in coordinating the mission with both the Israeli foreign ministry, local assets, and other FHs. All missions include a generator and moving equipment such as a forklift. The success of the team is in the flexibility of the staff. Board-certified physicians and nurses participate in erecting the FH structures and maintenance, including cleaning. The equipment is constantly being upgraded and the Israeli EMT-3 purchased equipment for purifying water and large inflatable tents. The time saved by having inflatable tents is invaluable in the setting of a disaster zone where time is critical.

### Ethical issues

Disaster medicine inherently involves complex ethical issues. Healthcare providers operating in mass casualty settings often face significant disparities between needs and available resources, necessitating adjustments in resource allocation and standards of care. This situation underscores the importance of transitioning from clinical ethics to public health ethics, which prioritize the common good over individual interests. Public health policies, especially during emergencies, may subordinate individual rights to population-level health outcomes, a concept that, while well understood, can be challenging for clinicians to implement.

Addressing ethical dilemmas in disaster-stricken areas is an ongoing process during the provision of care. Ethical codes and expectations specific to disaster response are well-documented in the literature and are regarded as integral to medical practice under such conditions. Major international organizations—such as the World Medical Association, the International Federation of Red Cross and Red Crescent Societies, and the American Medical Association— have published guidelines on ethics in disaster settings.

Common themes across these ethical standards emphasize distributive justice, including:

- Transparency in decision-making
- Consistent application of triage and access to care
- Accountability of decision-makers
- The recognition that access to healthcare is a fundamental human right

A widely used framework in medical ethics is the "Four Principles" approach proposed by Beauchamp and Childress which evaluates:

1. Respect for Autonomy: Honoring individuals’ rights to make their own choices based on personal values.
2. Justice: Ensuring equitable treatment and fair distribution of scarce resources.
3. Nonmaleficence: Obligation to avoid causing harm.
4. Beneficence: Acting in the best interest of patients to promote their welfare [38].

The international community has also emphasized the need to establish standards that protect the dignity of disaster victims. One such effort is the Sphere Standards, developed jointly by the International Committee of the Red Cross and Red Crescent Movement, and other non-governmental organizations, which outline minimum humanitarian response standards based on human rights principles [38]. These standards emphasize core values like accountability, transparency, and neutrality, recognizing potential conflicts among them, and advocating for adherence to codes of conduct to mitigate moral distress.

Mass-casualty triage, in particular, must be implemented when resources are insufficient for all patients. The guiding principle is to maximize overall benefit—saving the greatest number of lives with the limited resources available. This often shifts the focus toward utilitarian ethics, where prioritization is based on the likelihood of survival and potential outcomes [39].

The unique ethical challenges in disaster responses demand careful consideration. Addressing these issues proactively is vital for effective planning and delivery of care. Medical personnel must be equipped not only technically but also emotionally and ethically to confront the difficulties inherent in such scenarios.

### Wellness

Disaster response can result in morbidity to the staff in terms of both medical and psychological issues [40, 41]. Several strategies are intentionally introduced to reduce stress and mental issues among the staff. Every mission has a professional mental health worker, whether a psychiatrist, psychologist, or social worker. They are present for the patients as well as the staff. A refreshment area is built so that the staff can take coffee breaks. Departmental debriefings are conducted to improve medical issues, but also to help the staff ventilate and reflect. In addition, direct satellites are set up so that the staff can communicate with family back home [42].

In addition to medical clowns having a significant impact on patient care, they also have a reassuring effect on the staff as well. Medical clowning can help avoid deterioration of acute stress disorders and thus should not be considered “nice to have” but a “must have.” [24] (see Table 1).

**Table 1:** Lessons Learned from the Israel Defense Forces EMT-3 Field Hospital

	**Lesson Learned**
Hospital design	Flexibility in whether the setup should be a hybrid or stand-alone design
Emergency department	Ability to work independently before the entire field hospital is setup Importance of bringing standard equipment: monitors, video laryngoscopes, and ventilators Extensive use of point-of-care ultrasound to minimize radiation from X-rays Staff according to patient arrival (mostly during daylight hours as opposed to dark)
Surgical specialties	Most operations are for orthopedic injuries Most orthopedic procedures are for treatment of soft tissue injuries, wound closure, and external fixations
Pediatrics	Need to have a range of pediatric specialists: general pediatricians, pediatric emergency medicine specialists, and pediatric intensive care specialists Age-appropriate equipment Importance of improvising such as preparing customized fluids for premature infants from standard fluids or creating a makeshift incubator Separating pediatric from adult patients Importance of procedural sedation and medical clowns
Inpatient wards	Combined medical-surgical ward Staffed by internists, family medicine physicians, pediatricians
Intensive care unit	Can manage complex resource-intensive patients on-site Need to adapt clinical decision-making, optimize resource allocation, and deliver safe, effective care under austere conditions Build modular capacity (“model of four” beds) Maintain strict nurse team structures (2 + 2 per shift) to ensure safety and continuity
Obstetrics/Gynecology	Ob/gyn team manages complicated deliveries, cesarean sections, and pregnancy-related complications Provides critical preventive care and family planning services. This includes contraceptive counseling, prenatal care for pregnant women, and treatment of gynecological infections
Infection control	Important for coordinating ventilation, and isolation when necessary; potable drinking water; food safety; facilitation of hand hygiene; instrument cleaning and disinfection/sterilization; location of toilets and latrines; pest control; human and solid waste disposal, including sharps Importance of infectious disease specialist and infection control nurse
Nursing	Importance of maintaining nurse:patient ratio as described in the Bluebook Importance of quality nursing leadership Importance of versatility in terms of being able to work in different departments
Informatics	Importance of electronic emergency medical record Future advances may be to translate instructions into the patient’s native language
Laboratory services	Importance of blood tests and microbiology cultures
Radiology	PACS is well integrated throughout all departments Administering most X-rays in a dedicated radiology suite to minimize radiation exposure to staff and patients Integrating advanced point-of-care ultrasound
Logistics	Importance of electricians, civil engineers, and public health experts Generator and forklift are must haves Flexibility of all staff including physicians and nurses in helping with erecting the FH structures and routine maintenance Inflatable tents to save time setting up the field hospital

## Conclusion

The framework for the Israeli EMT-3 is the guidelines of the WHO as outlined in the Blue Book. The main lesson learned is that the success of the Israeli EMT-3 is based on professionalism and flexibility. The staff consists of highly trained professionals, including board-certified physicians and nurse specialists who convert their everyday skills into those used in the disaster setting. Flexibility includes having these professionals take on logistical responsibilites. Operational flexibility includes the last-minute decision to implement a hybrid versus a standalone model, using local supplies, and quickly adapting to an urgent need. While the lessons here are from the perspective of an EMT-3, they can be applied to other response teams to improve outcomes for disaster victims throughout the world.

## Data Availability

Availability of data and materials is upon reasonable request from the corresponding author.

## Abbreviations

ED: Emergency department
EMR: Electronic medical record
EMT: Emergency medical team
FH: Field hospital
ICU: Intensive care unit
IDF: Israel Defense Forces
IDF-FH: Israel Defense Forces field hospital
IPC: Infection prevention and control
OB/GYN: Obstetrics/Gynecology
PACS: Picture archiving and communication system
POCUS: Point-of-care ultrasound
WHO: World Health Organization
